# Mendelian randomisation analyses of UK Biobank and published data suggest that increased adiposity lowers risk of breast and prostate cancer

**DOI:** 10.1038/s41598-021-04401-6

**Published:** 2022-01-18

**Authors:** Hasnat A. Amin, Pimpika Kaewsri, Andrianos M. Yiorkas, Heather Cooke, Alexandra I. Blakemore, Fotios Drenos

**Affiliations:** Department of Life Sciences, College of Health, Medical and Life Sciences, Brunel University London, Kingston Lane, Uxbridge, UB8 3PH Middlesex UK

**Keywords:** Breast cancer, Cancer epidemiology, Cancer prevention, Risk factors, Genetic association study

## Abstract

Breast (BCa) and prostate (PrCa) cancer are the first and second most common types of cancer in women and men, respectively. We aimed to explore the causal effect of adiposity on BCa and PrCa risk in the UK Biobank and published data. We used Mendelian randomisation (MR) to assess the causal effect of body mass index (BMI), body fat percentage (BFP), waist circumference (WC), hip circumference (HC), and waist-to-hip ratio (WHR) on BCa and PrCa risk. We found that increased BMI, WC and HC decreased the risk of breast cancer (OR 0.70 per 5.14 kg/m^2^ [0.59–0.85, p = 2.1 × 10^–4^], 0.76 per 12.49 cm [60–0.97, p = 0.028] and 0.73 per 10.31 cm [0.59–0.90, p = 3.7 × 10^–3^], respectively) and increased WC and BMI decreased the risk of prostate cancer (0.68 per 11.32 cm [0.50–0.91, p = 0.01] and 0.76 per 10.23 kg/m^2^ [0.61–0.95, p = 0.015], respectively) in UK Biobank participants. We confirmed our results with a two-sample-MR of published data. In conclusion, our results suggest a protective effect of adiposity on the risk of BCa and PrCa highlighting the need to re-evaluate the role of adiposity as cancer risk factor.

## Introduction

Breast and prostate cancer are the most common and second most common types of cancer diagnosed worldwide in men and women, respectively^1^. In 2010, the combined cost of breast and prostate cancer to the NHS was £664 million^2^. The number of cases are expected to rapidly increase and, by 2040, are estimated to be 20.2% higher for breast cancer and 38.5% higher for prostate cancer in comparison to 2018^1^. Both cancer types are preventable in many cases, making robust identification of their modifiable risk factors important.

A recent campaign by Cancer Research UK^3^ has emphasised obesity as being a causal risk factor for cancer comparable to smoking. It has been proposed that the metabolic environment in obese people is conducive to oncogenic transformation^4^. However, previously published evidence on the relationship between adiposity and breast and prostate cancer does not consistently support this view^5–7^. It has been suggested that adiposity is a risk factor for breast cancer in post-menopausal women^8^, but not in pre-menopausal women^9^. A meta-analysis of 67 studies looking at the relationship between BMI and risk of prostate cancer recently showed that the relationship between BMI and prostate cancer is inconsistent^10^.

Contrary to the view of obesity as cancer risk factor, previously published evidence suggest that adipose tissue may play a role in safely storing harmful chemicals^11^. Persistent organic pollutant (POP) concentrations increase by 2–4% per kg of weight loss and remain elevated for up to 12 months after a weight loss intervention^12^. This may be one of the reasons behind the present inconsistency in the findings between BMI and breast and prostate cancer risk.

Assessment of what exposures are causal is not trivial: “correlation is not causation”. The vast majority of studies carried out to examine the impact of adiposity on breast and prostate cancer risk are observational and may be susceptible to confounding. Mendelian randomisation (MR) is a method that uses genetic variants associated with an exposure of interest, but not with any confounders, to assess the causal effect of the genetically predicted exposure on an outcome. In order for the method to provide reliable estimates of the causal effect, it is also assumed that the chosen instruments are not related to the outcome of interest independently of the exposure.

With this work, we aim to explore the causal effect of adiposity on breast and prostate cancer risk in the UK Biobank (UKB), a large prospective cohort study, and published data^13–16^. We also aim to use the rich phenotype data collected as part of the UKB study to identify variables, including chemical exposure, that may explain the observed relationship between adiposity and breast and prostate cancer risk.

## Methods

### Population and study design

The UK Biobank (UKB) is a large prospective cohort study including information and biological samples for approximately 500,000 individuals, recruited between 2006 and 2011. The 22 UKB assessment centres throughout England, Wales and Scotland, collected baseline data from the participants in the form of questionnaires, physical and cognitive tests and blood and urine samples^17^. The age range of the participants at the time of enrolment in the study was between 40 and 69 years of age, with a mean age of 56.63 years and 57.10 years in men and women, respectively. The use of the data for this study was approved by the UK Biobank access committee (application 44566) and data release was from April 2021. All research was performed in accordance with relevant guidelines/regulations, and informed consent was obtained from all participants by the UK Biobank.

### Genotyping

488,377 individuals had been genotyped for up to 812,428 variants using DNA extracted from blood samples on either the UKB Axiom array (438,427 participants) or the UK BiLEVE Axiom array (49,950 participants). Variants that did not pass standard quality control checks were excluded from any subsequent analyses in the UKB^18^. These included tests for the presence batch effects, plate effects, sex effects and array effects, as well as any departures from Hardy–Weinberg Equilibrium using a p-value threshold of 10^−12^—further details of these tests are available in the supplementary material provided by Bycroft et al.^18^ Variants with a minor allele frequency of < 0.01 and imputed variants with an INFO score of < 0.8 were excluded from any subsequent analyses in the UKB.

Sample genotyping quality control metrics were provided by UKB^18^. Samples were excluded from the analysis if they were outliers for missingness and/or PC-corrected heterozygosity and/or if they had any sex chromosome aneuploidies as well as if the genetically inferred sex differed from the reported sex. Samples which did not have a genetically determined White British ancestry were also excluded. A list of related individuals was also provided by UKB and one individual from each related pair was excluded at random.

### Phenotypes

We used data collected at baseline for body mass index (BMI, UKB field 21001), body fat percentage (BFP, UKB field 23099) from bio-impedance, waist circumference (WC, UKB field 48) and hip circumference (HC, UKB field 49). We calculated waist-to-hip ratio (WHR) by dividing WC by HC. The variables were standardised to a mean of 0 and a variance of 1.

We used cancer diagnoses information from the 1970s onward obtained from linkage to national cancer registries and health records (see http://biobank.ndph.ox.ac.uk/showcase/showcase/docs/CancerLinkage.pdf). Breast cancer cases are defined as females who have an ICD-10 code C50 recorded at least once (UKB field 40006). Prostate cancer cases are defined as males who have an ICD-10 code C61 recorded at least once (UKB field 40006). Females who have an ICD-10 code D05, for in situ carcinoma of the breast, without a C50 breast cancer entry were removed from the sample. Similarly, males with an ICD-10 code D075, for carcinoma in situ of prostate, without a C61 prostate cancer diagnosis were also removed from the sample.

Menopause information for females was obtained through the reported age of menopause information collected (UKB field 3581). This information was compared to the age of first breast cancer diagnosis to identify the pre- and post-menopausal cases. For women who did not have breast cancer, we used their menopause status at baseline to stratify them into pre- and post-menopausal. Women whose menopause status could not be determined were set to missing.

Exposure to chemicals was based on occupation exposure information. A participant was considered to have been exposed to chemicals frequently if they answered “Often” and/or “Sometimes” at least once for any of the following UKB fields: 22609 (Workplace very dusty); 22610 (Workplace full of chemical or other fumes); 22611 (Workplace had a lot of cigarette smoke from other people smoking); 22612 (Worked with materials containing asbestos); 22613 (Worked with paints, thinners or glues); 22614 (Worked with pesticides); and 22615 (Workplace had a lot of diesel exhaust).

### Statistical analyses

We used R 3.6.1^19^ to carry out analyses and generate plots, unless stated otherwise.

For the observational correlations, we removed prevalent cases and regressed the exposures (i.e. BMI, BFP, WC, HC and WHR) against prostate and breast cancer cases using a logistic regression adjusting for age at baseline.

We obtained genetic instruments for BMI, WC, HC and WHR from summary statistics from publicly available sex-stratified GWAS meta-analyses of European ancestry^15,16,20^. We included all independent variants that were associated with the trait as listed in the supplementary tables provided by the authors of the studies. Please see Supplementary Table S1 for a list of variants used.

The most recent GWAS of BFP that does not include UKB participants^20^ does not provide sex-stratified summary statistics and could not be used to obtain instruments for BFP. We therefore carried out association analyses for the 10 variants found to be associated with BFP by Lu et al.^20^ in the UKB using PLINK 2.0^21^, in males and females separately, and excluded those who had cancer to minimise any bias^22^. Please see Supplementary Table S1 for a list of independent variants associated with BFP along with their effect sizes.

We used the variants and their effect sizes (see Supplementary Table S1) to calculate additive genetic risk scores (GRSs) in the UKB using PLINK 2.0^21^. We regressed each GRS on its respective trait to obtain R^2^ and F-statistics (see Supplementary Table S2) to demonstrate that the instruments used are associated with their respective traits.

We carried out a one-sample MR in the UKB to assess the effect of the adiposity measures on breast and prostate cancer using the two-stage method^23^. In the first step, we regressed the adiposity measure on its respective GRS to obtain a set of fitted values for the adiposity measure. In the second step, we regressed the cancer outcome (i.e. breast cancer or prostate cancer) on the fitted values using a logistic regression, adjusted for age at baseline. The confidence intervals were obtained using bootstrap with 10,000 repetitions.

To test whether exposure to workplace chemicals or menopause status modified the causal effect of adiposity on breast and prostate cancer, respectively, we stratified the sample by menopause status and by chemical exposure and repeated the one-sample MR analyses described above. We then used the metagen() function from the meta package in R^24^ to perform a chi-squared test for heterogeneity between the strata.

It is assumed that the instruments selected for MR analyses are not related to the outcome of interest independently of the exposure (this is known as the “exclusion restriction” assumption). To test this assumption, we regressed the cancer outcome on each of the GRSs using a logistic regression, adjusted for the respective adiposity measure. If the assumption is valid, the GRS will not be associated with the outcome. Evidence of association means that the estimate effect may be affected by bias and methods such as MR Egger will provide a more accurate estimate of the causal effect.

To confirm any findings from our one-sample MR analyses, we used the TwoSampleMR package in R^25^ to perform a two-sample MR analysis using the inverse-variance weighted method with a multiplicative random effects model. We used the MR-Egger method as a sensitivity analysis and to detect the presence of pleiotropy (indicated by a statistically significant intercept term). The genetic instruments for the adiposity measures were obtained as described previously. We used the extract_outcomes_data(…,rsq = 1) function to extract the effect sizes and standard errors for the outcomes from Michailidou et al.^13^ and Shumacher et al.^14^, which are the meta-analysis of breast cancer GWASs (122,977 cases, 105,974 controls) and the meta-analysis of prostate cancer GWASs (79,194 cases, 61,112 controls), respectively. Please see Supplementary Table S3 for all variants used in the two-sample MR analyses along with their harmonised effect sizes.

To determine whether the associations of adiposity measures were independent of each other and of childhood adiposity, we used an extension of the MR method, known as multivariable MR (MVMR)^26^. MVMR is useful when the genetic instruments used are associated with more than a single risk factor tested, as in this case of overlapping SNPs for the adiposity measures. Instruments used in the MVMR for childhood adiposity were extracted from Vogelezang et al.^27^ We used MVMR as implemented in the mv_multiple() function from the TwoSampleMR package^25^, which also removes SNPs of LD > 0.001 between measures.

To explore the possibility of a non-linear causal relationship between adiposity and cancer risk, we removed prevalent cases and performed non-linear MR using the sliding window method described by Burgess et al.^28^ using a window size of 50,000 and a step size of 1000. The residuals that were used to order the data were obtained by regressing the adiposity measure on its genetic risk score, adjusted for age, age-squared^2^(to adjust for the non-linear effects of age), the first four genetic principal components (to account for population stratification) and genotyping array.

To identify potential confounders responsible for the observed positive association between BMI and incident breast cancer cases, we developed an algorithm that used a step-wise procedure to test which variables can minimise the effect size of BMI on breast cancer when added in the model as covariates. Only variables with more than 1000 non-missing observations associated with both BMI and breast cancer (p < 0.05) were considered. Categorical phenotypes were converted to separate binary variables. Age at baseline was included in all models.

### Ethics approval and consent to participate

UK Biobank has ethical approval from the North West Multi-centre Research Ethics Committee (16/NW/0274). The work was carried out under UK Biobank application 44566.

## Results

### Population characteristics

After QC, we had 177,471 females and 154,453 males remaining in the sample. Table 1 summarises their age, adiposity measures and lifetime smoking status based on the information collected at baseline.

**Table 1:** UK Biobank population characteristics by cancer status.

	Females			Males		
	All	Cases	Controls	All	Cases	Controls
N	177,471	9613	167,858	154,453	6817	147,636
Ever smoked (%)	55.71	57.69	55.60	65.03	66.2	64.98
Age (years)	56.63 (7.90)	58.93 (7.10)	56.49 (7.92)	57.10 (8.08)	62.99 (4.89)	56.83 (8.10)
BMI (kg/m^2^)	27.02 (5.14)	27.27 (4.93)	27.00 (5.15)	27.82 (4.23)	27.60 (3.83)	27.83 (4.24)
WC (cm)	84.56 (12.49)	85.71 (12.13)	84.50 (12.51)	97.03 (11.32)	97.27 (10.55)	97.02 (11.36)
HC (cm)	103.36 (10.31)	104.01 (9.92)	103.32 (10.33)	103.53 (7.58)	103.27 (7.04)	103.55 (7.61)
WHR (cm/cm)	0.82 (0.07)	0.82 (0.07)	0.82 (0.07)	0.94 (0.06)	0.94 (0.06)	0.94 (0.07)
BFP (%)	36.56 (6.87)	37.13 (6.53)	36.52 (6.89)	25.27 (5.80)	25.73 (5.57)	25.25 (5.80)

Sex-specific distribution of age and adiposity measures and lifetime smoking status in the full sample, in those who have breast/prostate cancer and in those who do not have breast/prostate cancer. Standard deviations are provided in round brackets, if applicable. N = sample size.

### Effect of observed adiposity on incident cancer risk

**Figure 1:**
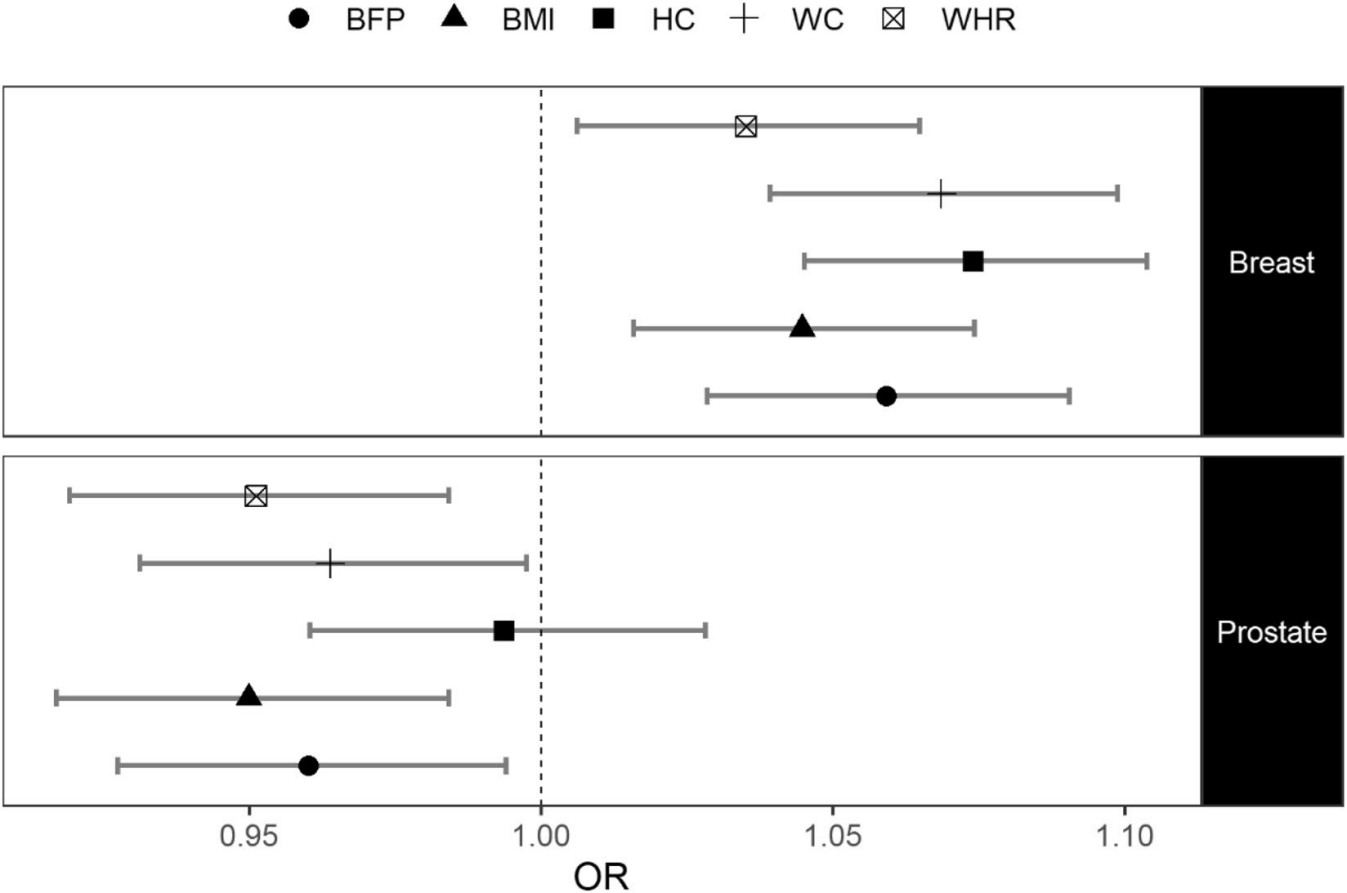
Effect of observed adiposity on incident cancer risk. Odds ratios per SD increase (OR) and 95% confidence intervals when regressing incident breast and prostate cancer cases on the adiposity measures using a logistic regression. *BMI* body mass index, *BFP* body fat percentage, *HC* hip circumference, *WC* waist circumference, *WHR* waist-to-hip ratio.

We first sought to examine the observational effect of the adiposity measures (i.e. BMI, BFP, WC, HC and WHR) on the risk of breast and prostate cancer. We only used incident cases to avoid a previous cancer diagnosis affecting any of the measures considered. We found that, in our sample, each of the adiposity measures are associated with an increased risk of breast cancer but, with the exception of HC, are associated with a decreased risk of prostate cancer (Fig. 1 & Supplementary Table S4).

### Causal effect of adiposity on cancer risk: one-sample MR

**Figure 2:**
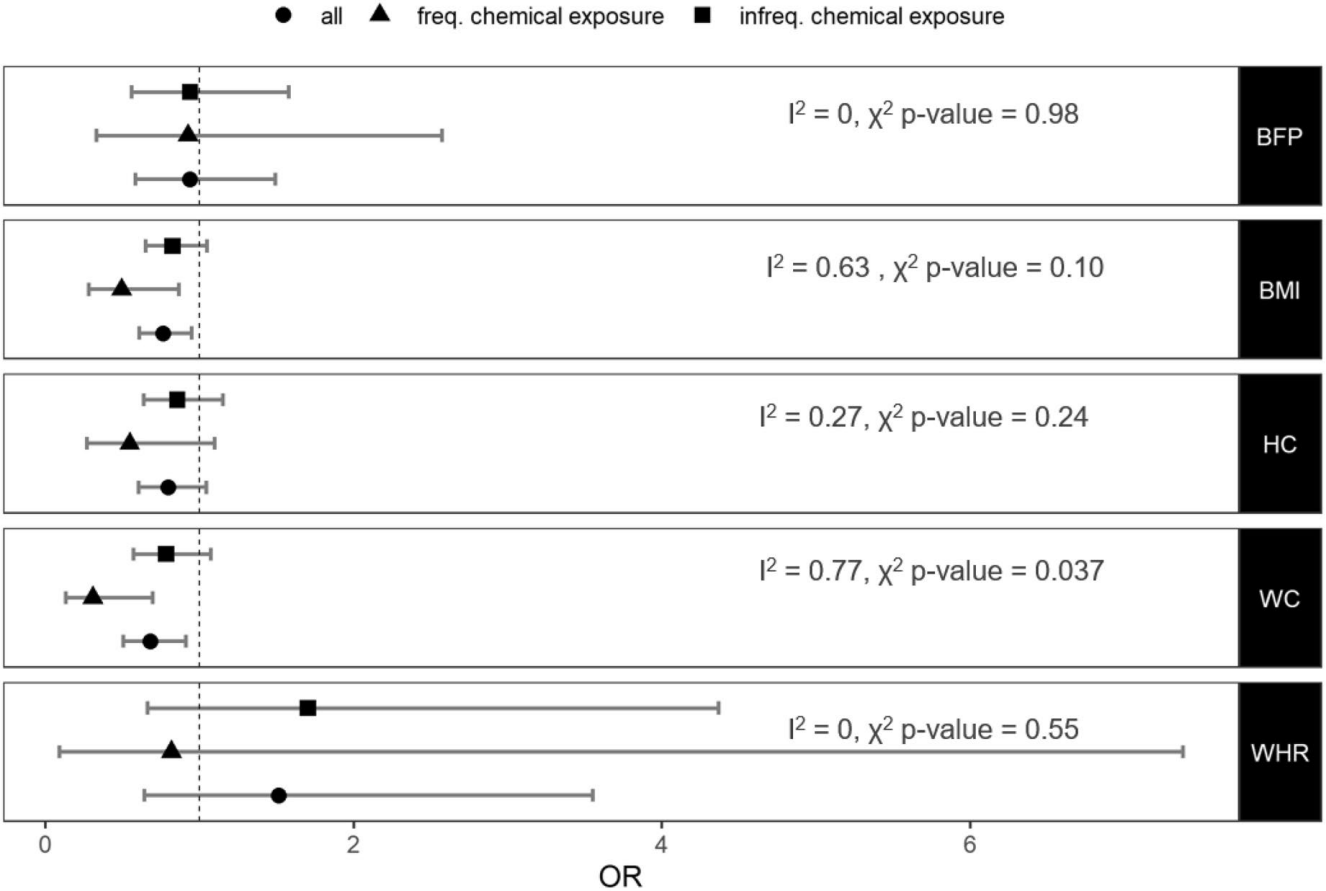
Causal effect of adiposity on prostate cancer. Odds ratios (OR) and 95% confidence intervals from a one-sample MR analysis of the causal effect of adiposity on prostate cancer risk in the complete sample (all) and in the selected strata. *BMI* body mass index, *BFP* body fat percentage, *HC* hip circumference, *WC* waist circumference, *WHR* waist-to-hip ratio.

**Figure 3:**
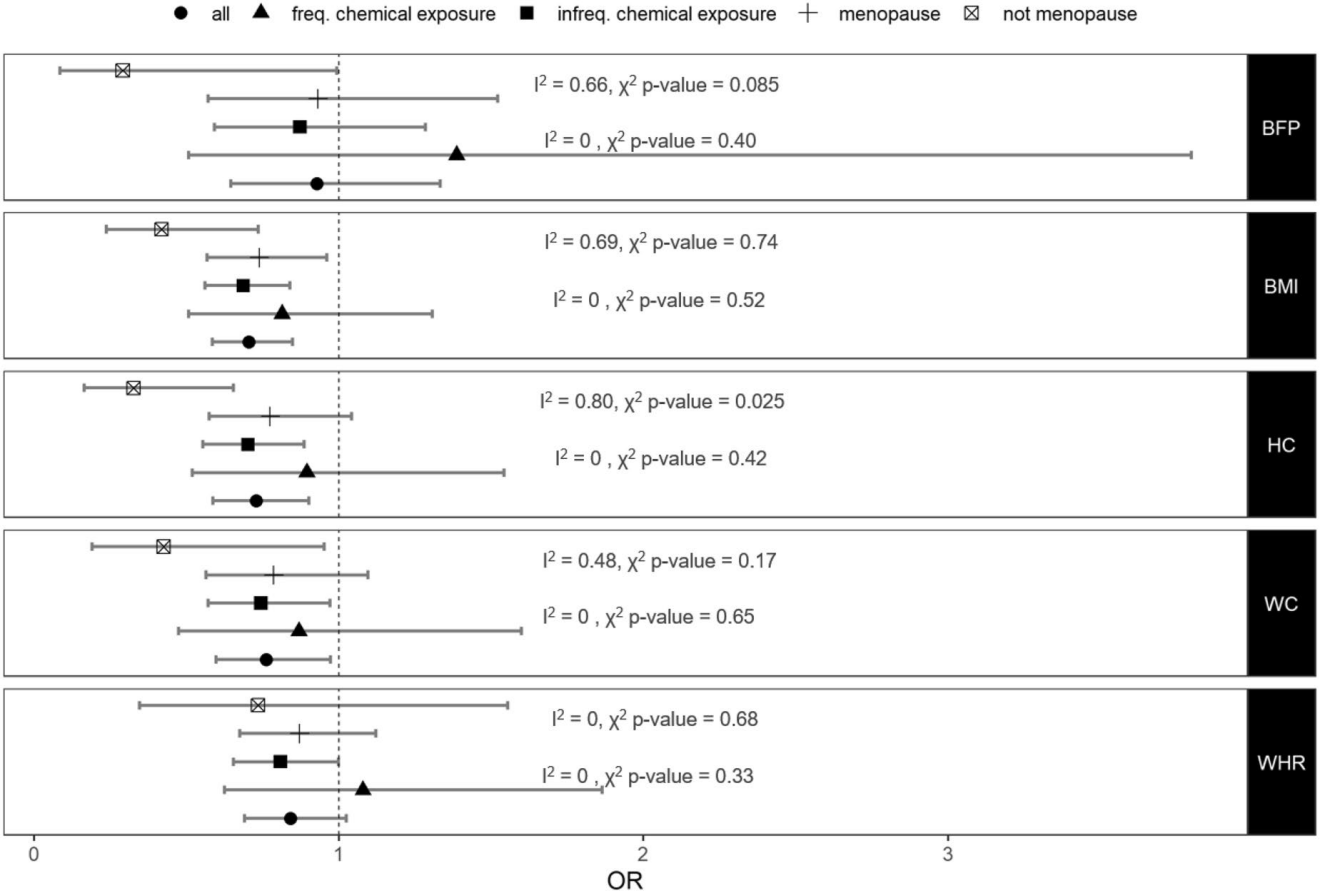
Causal effect of adiposity on breast cancer. Odds ratios (OR) and 95% confidence intervals from a one-sample MR analysis of the causal effect of adiposity on breast cancer risk in the complete sample (all) and in the selected strata. *BMI* body mass index, *BFP* body fat percentage, *HC* hip circumference, *WC* waist circumference, *WHR* waist-to-hip ratio. The top I^2^ values refer to the test of heterogeneity between pre- and post-menopausal cases. The bottom I^2^ values refer to the test of heterogeneity between those reporting frequent exposure to workplace chemical in comparison to those that report infrequent exposure.

We obtained estimates (odds ratios (OR) per SD unit increase) of the causal effect of the adiposity measures on breast and prostate cancer using one-sample MR (Figs. 2 & 3 and Supplementary Table S5). The variants that we used to generate the GRSs are available in Supplementary Table S1. The strength of the association between these GRSs and the adiposity measures are provided in Supplementary Table S2.

We found that increased BMI, WC and HC decreased the risk of breast cancer (OR 0.70 [95% CIs 0.59–0.85, p = 2.1 × 10^–4^], OR 0.76 [95% CIs 0.60–0.97, p = 0.028] and OR 0.73 [95% CIs 0.59–0.90, p = 3.7 × 10^–3^], respectively) and increased WC and BMI decreased the risk of prostate cancer (OR 0.68 [95% CIs 0.50–0.91, p = 0.01] and OR 0.76 [95% CIs 0.61–0.95, p = 0.015], respectively) based on our one sample MR using the UK Biobank individual level data.

### Testing the exclusion restriction assumption

We found that the associations between the GRSs for BMI, WC, HC and breast cancer were present even after adjusting for BMI, WC, and HC, respectively (Supplementary Table S6). This suggests that these GRSs violate the exclusion restriction assumption, i.e. the genetic instrument may affect the outcome independently of the exposure and the estimated causal effect may be biased. We found that the GRS for BMI was not associated with prostate cancer independently of BMI. However, the GRS for WC was associated with prostate risk independently of WC (Supplementary Table S6), so the estimate of the causal effect of WC on prostate cancer risk obtained in the UKB may also be biased. We addressed these issues by confirming our one-sample MR results using the two sample MR Egger method in published data as described below, which is robust even when the exclusion restriction assumption is violated.

### Using two-sample MR to confirm the one-sample MR results

We sought to replicate our findings using external outcome summary statistics from meta-analyses of 122,977 breast cancer cases^13^ and 79,194 prostate cancer cases^14^, and external exposure summary statistics from Lu et al.^20^ and GIANT^15,16^. We used two-sample MR to assess the causal effect of adiposity measures on breast and prostate cancer risk (Table 2). We found that increased BMI, WC, HC, and BFP are causally protective for breast cancer using the inverse variance weighted method. The p-values of the intercept from the MR Egger method suggest that the instruments used for BMI, WC, and HC may be pleiotropic, but the causal estimates generated using MR Egger show that increased BMI, WC, and HC are still protective for breast cancer when pleiotropy is considered (Table 2). We also found that BMI and WC were protective for prostate cancer.

**Table 2:** Two-sample MR estimates of the causal effect of adiposity on breast and prostate cancer.

Cancer	Exposure	Method	OR [95% CIs]	p	p^(intercept)^
Breast	BMI	MR Egger	0.55 [0.36–0.83]	7.14E − 03	1.41E − 01
Breast	BMI	Inverse variance weighted	0.74 [0.65–0.85]	1.36E − 05	NA
Breast	BFP	MR Egger	0.30 [0.14–0.62]	1.19E − 02	1.48E − 01
Breast	BFP	Inverse variance weighted	0.51 [0.34–0.74]	4.55E − 04	NA
Breast	HC	MR Egger	0.28 [0.13–0.59]	3.99E − 03	2.33E − 02
Breast	HC	Inverse variance weighted	0.69 [0.52–0.90]	6.99E − 03	NA
Breast	WC	MR Egger	0.36 [0.20–0.66]	4.62E − 03	2.65E − 02
Breast	WC	Inverse variance weighted	0.74 [0.61–0.91]	3.23E − 03	NA
Breast	WHR	MR Egger	0.73 [0.18–3.03]	6.73E − 01	8.91E − 01
Breast	WHR	Inverse variance weighted	0.81 [0.62–1.05]	1.15E − 01	NA
Prostate	BMI	MR Egger	0.79 [0.50–1.26]	3.31E − 01	9.87E − 01
Prostate	BMI	Inverse variance weighted	0.79 [0.70–0.90]	2.91E − 04	NA
Prostate	BFP	MR Egger	0.56 [0.27–1.18]	1.66E − 01	3.03E − 01
Prostate	BFP	Inverse variance weighted	0.82 [0.59–1.13]	2.21E − 01	NA
Prostate	HC	MR Egger	0.83 [0.28–2.52]	7.53E − 01	9.14E − 01
Prostate	HC	Inverse variance weighted	0.79 [0.60–1.03]	7.82E − 02	NA
Prostate	WC	MR Egger	1.15 [0.26–5.11]	8.59E − 01	6.07E − 01
Prostate	WC	Inverse variance weighted	0.77 [0.61–0.98]	3.31E − 02	NA
Prostate	WHR	MR Egger	0.46 [0.17–1.19]	2.49E − 01	3.33E − 01
Prostate	WHR	Inverse variance weighted	0.83 [0.64–1.07]	1.52E − 01	NA

Odds ratios (OR) and 95% confidence intervals from a two-sample MR analysis of the causal effect of adiposity on breast and prostate cancer risk in women and men, respectively. p^(intercept)^ = p-value of the intercept from the MR Egger method.

*BMI* body mass index, *BFP* body fat percentage, *HC* hip circumference, *WC* waist circumference, *WHR* waist-to-hip ratio.

### Testing for the presence of non-linearity

Both one-sample MR and two-sample MR assume that the exposure-outcome relationship is linear, but this is not always true. We therefore carried out non-linear MR analyses to visualise the association between the adiposity measures and the outcomes in different ranges of the exposure (please see Supplementary Fig. S1 for the sliding window plots). There appears to be visual evidence of non-linearity in the relationship between adiposity and breast cancer, but no such pattern is evident for the relationship between adiposity and prostate cancer.

### Assessing the independent contribution of different adiposity measures to cancer risk

We next performed two-sample multivariable MR to identify whether the protective effects of increased BMI, WC, HC and BFP on breast cancer risk are independent of each other (Supplementary Table S7). We found that BMI was still protective for breast cancer independently of all other measures. We also found that BMI was still protective for prostate cancer independently of all other measures.

### Assessing the independent effects of childhood and adult adiposity on cancer risk

We carried out a two-sample multivariable MR to assess whether the effect of the adiposity measures on breast and prostate cancer was independent of childhood BMI (Supplementary Table S8). We found that none of the adiposity measures associated with breast cancer independently of childhood BMI. We also found that BMI and WC were protective for prostate cancer independently of childhood BMI.

### Effect of adiposity on breast cancer by menopause status

Since BMI has shown to be protective for breast cancer in pre-menopausal women, we stratified women based on their menopause status at breast cancer diagnosis (or at baseline for controls) and repeated the one-sample MR analyses. We found that the protective effect of adiposity on breast cancer was stronger in pre-menopausal women in comparison to post-menopausal women (Fig. 3).

### Effect of adiposity on cancer risk by occupational chemical exposure

We hypothesised that the protective effect of adiposity may be due to adipose tissue absorbing and safely storing environmental carcinogens. We, therefore, stratified our sample, based on self-reported exposure to dust and/or chemicals and/or fumes at work and repeated the analyses. We found that the protective effect of increasing adiposity on prostate cancer was stronger in men who reported that they were frequently exposed to potentially hazardous substances at work in comparison those who were not (Fig. 2). There is no statistically significant heterogeneity (chi-squared p-value > 0.1) in the effect sizes between women who reported that they were frequently exposed to potentially hazardous substances at work and those who did not (Fig. 3).

### Identification of confounders of the BMI-breast cancer association

We used a stepwise procedure to identify any confounding variables that might explain the opposite direction of effects estimated by the observational and MR associations of BMI with risk of breast cancer. Supplementary Table S9 lists the fields that were both associated with incident breast cancer risk and attenuated the detrimental effect of BMI. The ln(OR) of breast cancer per SD unit increase in BMI is reduced tenfold when ankle spacing width, frequency of stair climbing, amount of moderate physical activity, macular degeneration and leukocyte count are added to the model, but it does not decrease below zero. It is possible that the variables our algorithm selects may be associated with a missing or currently unknown higher order variable that may explain the discrepancy between the observed and causal associations between BMI and risk of breast cancer.

## Discussion

We sought to assess the causal effects of increased adiposity on the risk of breast and prostate cancer. We found that increased adiposity measures were associated with a lower risk of incident prostate cancer, but with an increased risk of incident female breast cancer. Both one-sample MR and two-sample MR analyses showed that increased BMI and WC were protective for both breast and prostate cancer and that HC was protective for breast cancer. Multivariable MR analyses suggest: that BMI is the independent driver of these protective associations; and that childhood BMI attenuates the association between adiposity and breast cancer, but not between adiposity and prostate cancer. Stratified analyses suggest that the protective effect of adiposity on breast cancer and on prostate cancer may be enhanced in pre-menopausal women and in men exposed to workplace chemicals, respectively. When we attempted to identify the confounders responsible for the observed detrimental association between increasing BMI and breast cancer, we found a number of variables that may be involved, but these are of a currently uncertain clinical significance.

In this work, we found that increases in all of the adiposity measures we tested were observationally associated with a higher number of breast cancer cases. In this respect, the UK Biobank is in agreement with a previously published large meta-analysis of 126 studies finding the same association^29^. The inverse associations between the adiposity measures and prostate cancer were more surprising. Here the evidence are more heterogeneous, as illustrated by a recent large scale meta-analysis^10^, which found an overall null association between BMI and prostate cancer, but found an inverse association between BMI and prostate specific antigen concentrations. Furthermore, a number of well powered studies^30,31^ have also identified an inverse association between BMI and prostate cancer, so our results in the UK Biobank are, therefore, not unusual. Furthermore, increased adiposity has been suggested as protective for low grade prostate cancer^10^, the prostate cancer cases in the UKB are likely to be low grade due to the age of the sample, and this may further explain why we found an inverse observational relationship between adiposity and prostate cancer risk.

Since observational studies cannot directly provide information on cause-and-effect relationships, we carried out MR analyses to see whether the associations we found were causal. We found that adiposity was causally protective for breast cancer and our results are similar to those reported by Guo et al.^6^ using the same study but with a lower number of cases. Observationally, adiposity is reportedly protective for pre-menopausal breast cancer^9^ and our MR results, which show that the protective effect is stronger in pre-menopausal women, are in agreement with this. Considering all adiposity measures together, we found that BMI, a body mass measure, appears to drive the causal effect over the adiposity measures of body shape. We also show that, whilst BMI may explain the association between the other adiposity measures and breast cancer, childhood BMI may in fact explain the association between adulthood BMI and breast cancer—the latter is in agreement with Richardson et al.^32^ In these cases the continuity of the BMI phenotype throughout life fits well with the model of life-long exposure but also makes the interpretation of the results more difficult. It is possible that for breast cancer, childhood BMI is a better proxy of female body adiposity excluding breast fat and that this “core” adipose tissue is responsible for the protective effect seen here.

We also carried out MR analyses for prostate cancer. We found that BMI and WC are causally protective for prostate cancer in the UK Biobank dataset and this is supported by our analysis of external data. Davies et al.^7^ report no causal effect of BMI on prostate cancer, but this may be due to lack of statistical power because they used a smaller number of cases (20,848 vs. 79,194). Our multivariable MR results suggest that the protective association between WC and prostate cancer is driven again by BMI, a body mass measure, and that the association between BMI and WC is not attenuated by childhood BMI.

There is previously published evidence which suggests that adipose tissue may play a role in safely storing harmful chemicals^11^. Persistent organic pollutant (POP) concentrations increase by 2–4% per kg of weight loss and remain elevated for up to 12 months after a weight loss intervention^12^. We hypothesised that the protective effect of increasing adiposity on prostate and breast cancer risk might be explained by its ability to sequester potentially carcinogenic substances. Our results, which show that the protective effect was enhanced in men reporting more frequent exposure to potentially carcinogenic substances at work, support our hypothesis in prostate cancer. The same effect was not observed in female breast cancer, which may be due to an insufficient number of cases or due to a more complex underlying mechanism. In both breast and prostate cancer cases, it appears that the overall mass of adipose tissue, as measured by BMI, is more relevant than either % body fat or where the fat accumulates, measured by the WHR, WC and HC. This fits well with the idea of adipose tissue operating as a sink to store potentially harmful chemicals, but a more detailed investigation where exposure is accurately measured is required.

The direction of the observational association between BMI and breast cancer is opposite to that of the causal effect, which suggests that the former is confounded. We found that variables relating to physical activity (i.e. frequency of stair climbing and moderate physical activity) may be one source of confounding and this is supported by the fact that increased physical activity is protective for breast cancer^33^. Our algorithm also selected macular degeneration (an eye disease for which increasing age is the strongest risk factor and circulating lipids have also been involved)^34^, ankle width (which might represent swelling of the lower extremities—symptoms of diabetes and cardiovascular disease), and leukocyte count (a marker of systemic inflammation)^35^. These variables are likely to represent a currently undefined higher order variable, perhaps biological age or a marker of overall health, and further investigation is required to identify what this variable might be and whether or not it can be modified to minimise breast cancer risk.

A number of limitations are present in our work. The UK Biobank study, despite its sample size and almost comprehensive phenotyping, does have a "healthy volunteer" selection bias. The rate of cancer is lower in comparison to the general population^36^. Also, the proportion of adults who were overweight or obese among men and women in the UK population was 78% and 73%, respectively, compared to 74% and 60%, respectively, for the same age group in the UK Biobank^37^. The sample is, therefore, not representative of adiposity in the wider UK population. The difficulty in measuring adiposity should also be mentioned. The adiposity measures we use consider different aspects of adiposity.

## Conclusions

In conclusion, we found that increased adiposity is causally protective for breast and prostate cancer and the effects in the prostate cancer may be modified by exposure to potentially carcinogenic substances. Further work needs to be done to identify variables that are responsible for the observed relationship between increased BMI and increased risk of breast cancer. It is clear that reduction of adiposity, in and of itself, may not reduce the risk of breast and prostate cancer as the recent campaign by Cancer Research UK^3^ might suggest. As adiposity is a known risk factor for other age-related diseases, such as type-2-diabetes and cardiovascular disease, it is necessary to explore the mechanisms through which adiposity may protect against certain types of cancer and to identify how the former can be minimised without sacrificing the latter.

## Supplementary Information

Supplementary Information.

## Data Availability

This research has been conducted using the UK Biobank Resource under project 44566 (https://www.ukbiobank.ac.uk/2018/12/genetic-and-non-genetic-factors-able-to-predict-and-modify-the-risk-of-different-types-of-cancer/). All bona fide researchers can apply to use the UK Biobank resource for health-related research that is in the public interest.
