# Supplementary material for: Mendelian randomisation analyses of UK Biobank and published data suggest that increased adiposity lowers risk of breast and prostate cancer
Source: Sci Rep. 2022 Jan 18;12:909. doi: 10.1038/s41598-021-04401-6 (PMC8766553; doi:10.1038/s41598-021-04401-6)

Supplementary Figure 1

Non-linear MR analyses between the adiposity measures tested and the outcomes in different ranges of the exposure using a sliding window approach. The x-axis is the median of the measure in the window and the log(OR) of the outcome is on the y-axis

Panel I: Breast cancer

Body fat %

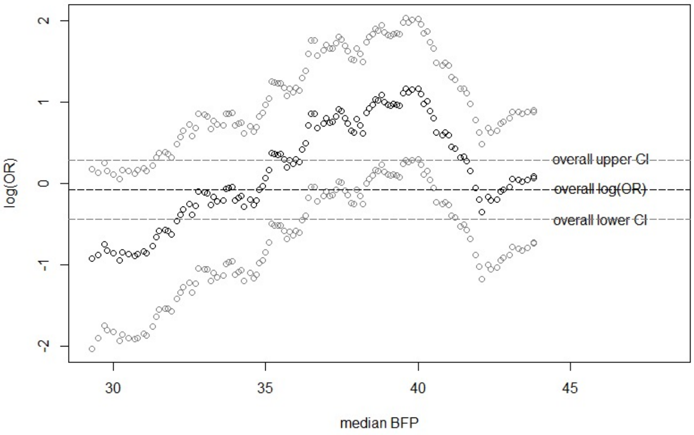

BMI

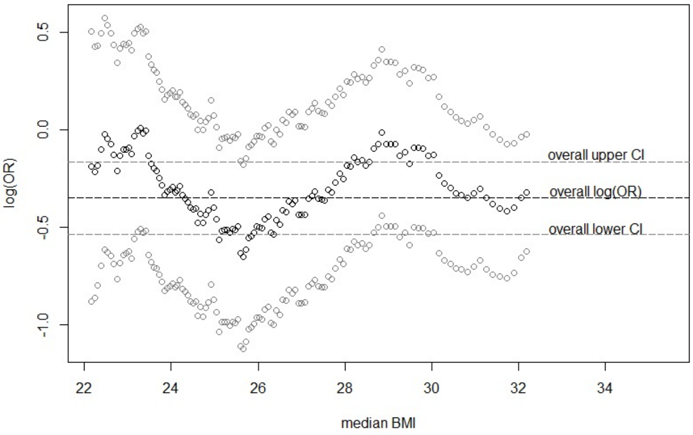

Hip circumference

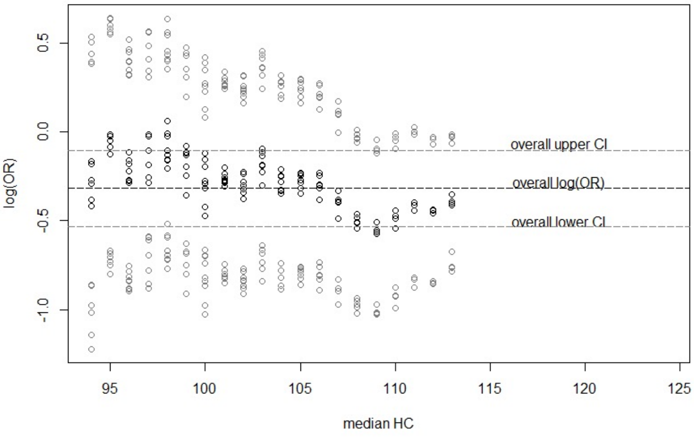

Waist circumference

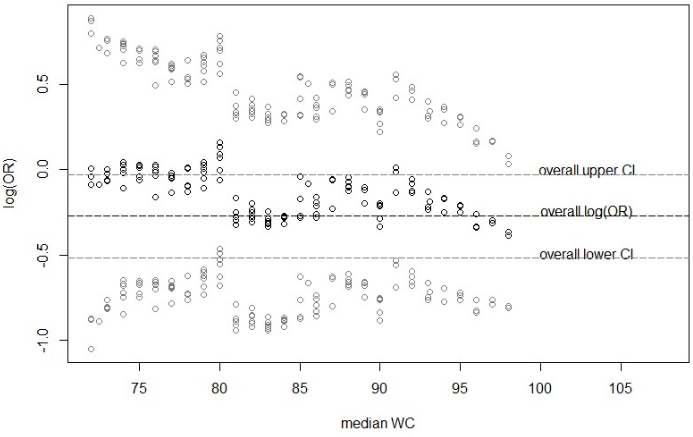

Wait to hip ratio

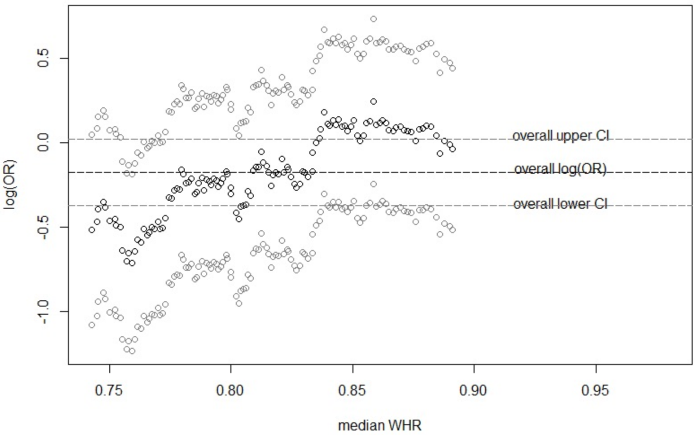

Supplementary Figure 1

Non-linear MR analyses between the adiposity measures tested and the outcomes in different ranges of the exposure using a sliding window approach. The x-axis is the median of the measure in the window and the log(OR) of the outcome is on the y-axis

Panel II: Prostate cancer

Body fat %

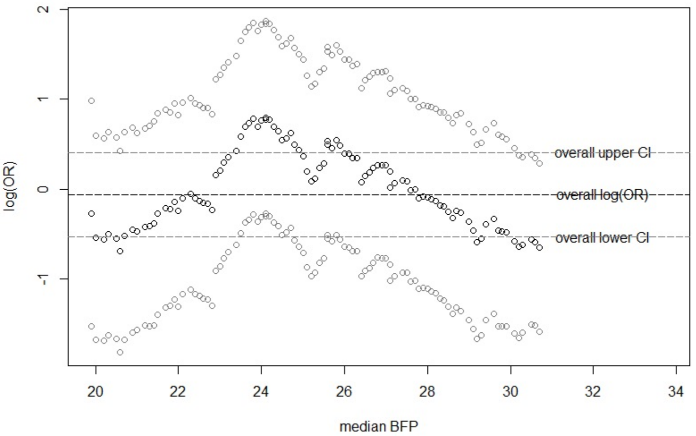

BMI

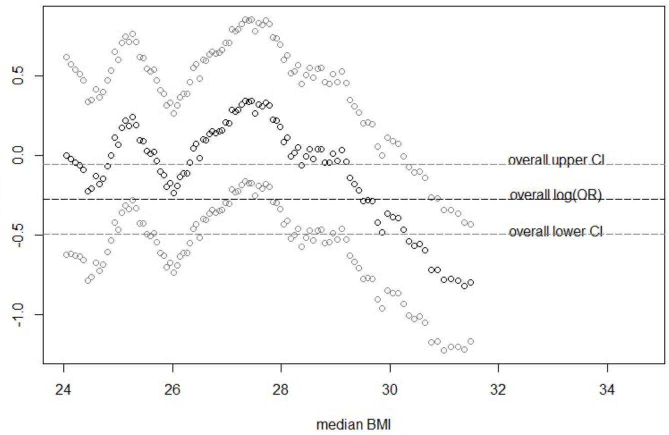

Hip circumference

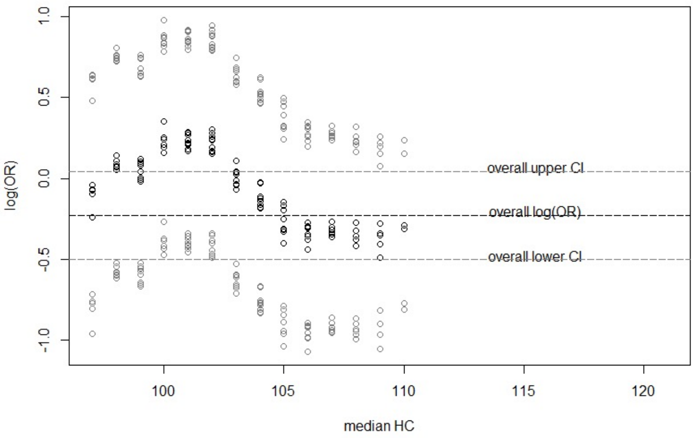

Waist circumference

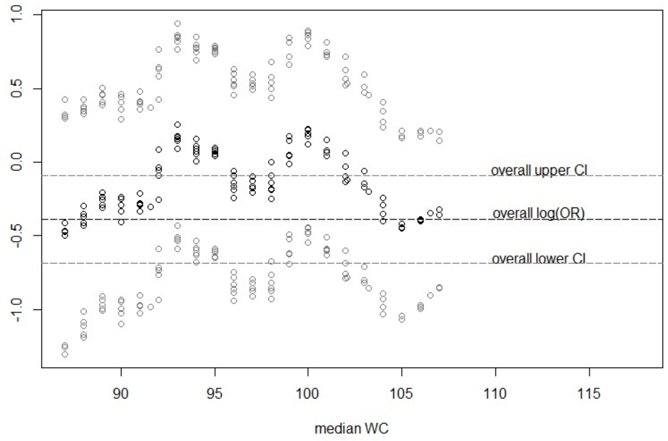

Wait to hip ratio

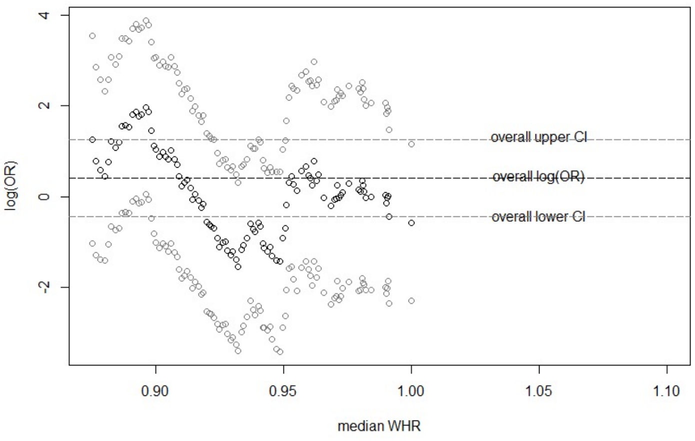

Supplement: Supplementary file 1 — Supplementary Information. [file 41598_2021_4401_MOESM1_ESM.zip › Supplementary/Supplementary_Figures/SFigure_1.pdf]
